# *N*-Nonyloxypentyl-l-Deoxynojirimycin Inhibits Growth, Biofilm Formation and Virulence Factors Expression of *Staphylococcus aureus*

**DOI:** 10.3390/antibiotics9060362

**Published:** 2020-06-26

**Authors:** Eliana De Gregorio, Anna Esposito, Adriana Vollaro, Maria De Fenza, Daniele D’Alonzo, Antonella Migliaccio, Vita Dora Iula, Raffaele Zarrilli, Annalisa Guaragna

**Affiliations:** 1 Department of Molecular Medicine and Medical Biotechnology, University of Naples Federico II, Via S. Pansini 5, 80131 Naples, Italy; vollaroadriana@libero.it; 2 Department of Chemical Sciences, University of Naples Federico II, Via Cintia, 80126 Naples, Italy; anna.esposito5@unina.it (A.E.); maria.defenza@unina.it (M.D.F.); daniele.dalonzo@unina.it (D.D.); 3 Department of Public Health, University of Naples Federico II, Via S. Pansini 5, 80131 Naples, Italy; antonella.migliaccio10@gmail.com (A.M.); rafzarri@unina.it (R.Z.); 4 Complex Operative Unit of Clinical Pathology, “Ospedale del Mare-ASL NA1 Centro”, 80131 Naples, Italy; dora.iula@gmail.com

**Keywords:** antimicrobial agents, antibiofilm agents, anti-virulence agents, deoxynojirimycin (DNJ), *Staphylococcus aureus*, iminosugars

## Abstract

*Staphylococcus aureus* is one of the major causes of hospital- and community-associated bacterial infections throughout the world, which are difficult to treat due to the rising number of drug-resistant strains. New molecules displaying potent activity against this bacterium are urgently needed. In this study, d- and l-deoxynojirimycin (DNJ) and a small library of their *N*-alkyl derivatives were screened against *S. aureus* ATCC 29213, with the aim to identify novel candidates with inhibitory potential. Among them, *N*-nonyloxypentyl-l-DNJ (l-NPDNJ) proved to be the most active compound against *S. aureus* ATCC 29213 and its clinical isolates, with the minimum inhibitory concentration (MIC) value of 128 μg/mL. l-NPDNJ also displayed an additive effect with gentamicin and oxacillin against the gentamicin- and methicillin-resistant *S. aureus* isolate 00717. Sub-MIC values of l-NPDNJ affected *S. aureus* biofilm development in a dose-dependent manner, inducing a strong reduction in biofilm biomass. Moreover, real-time reverse transcriptase PCR analysis revealed that l-NPDNJ effectively inhibited at sub-MIC values the transcription of the *spa*, *hla*, *hlb* and *sea* virulence genes, as well as the *agrA* and *saeR* response regulator genes.

## 1. Introduction

*Staphylococcus aureus* is an important human pathogen responsible for a variety of community- and healthcare-associated infections [1,2], which are difficult to treat and to eradicate because of the acquisition of multiple antibiotic resistance [3] and biofilm growth of this organism on implantable medical devices [4]. *S. aureus* produces several virulence factors, such as adhesins [5,6], secreted toxins and extracellular enzymes [7,8,9], which contribute to biofilm growth and host damage during infection [7,8,9]. The rapid emergence of increasingly resistant *S. aureus* strains [1,2,3] has led to the necessity to develop novel therapeutic agents against persistent infections caused by this organism [1,4,10,11]. 

**Figure 1:**
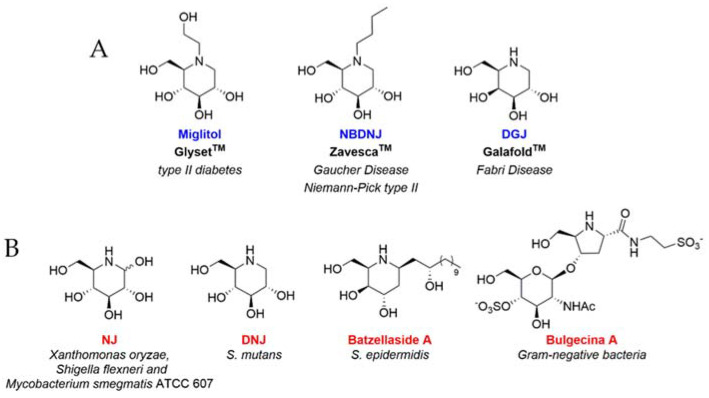
(**A**) Iminosugar-based drugs on the market. (**B**) Iminosugars showing antibacterial activity.

Iminosugars are natural or synthetic sugar analogues having an amino function in place of the endocyclic oxygen of the corresponding carbohydrate [12]. Due to their excellent ability to inhibit and/or enhance the catalytic properties of ubiquitous carbohydrate processing enzymes, such as glycosidases and glycosyltransferases, iminosugars represent the most promising class of glycomimetics, exhibiting broad-spectrum therapeutic potential [13,14]. Three iminosugar-based drugs (Figure 1A) are currently marketed for treating type II diabetes (Glyset^®^) and lysosomal storage disorders, including Gaucher and Niemann–Pick diseases type C (Zavesca^®^, *N*-butyl- deoxynojirimycin, NBDNJ) and Fabry diseases (Galafold^®^, deoxygalactonojirimycin, DGJ) [12,13,15]. In addition, a variety of other iminosugars have been proposed as therapeutic candidates against malignancies [16], viral infections [17,18] and other genetic disorders, including cystic fibrosis [19,20,21,22]. Conversely, iminosugars have displayed only in a few cases efficacy against bacterial pathogens (Figure 1B). Nojirimycin (NJ) exhibited antibacterial activity against *Xanthomonas oryzae*, *Shigella flexneri* and *Mycobacterium smegmatis* ATCC 607 [23], while deoxynojirimicyn (DNJ) was found to inhibit *Streptococcus mutans* biofilm formation [24,25,26]. Other modified iminosugars such as Batzellaside A and Bulgecin A held interesting properties against *Staphylococcus epidermidis* and Gram-negative bacteria, respectively [23]. Eventually, selected piperidine and indolizidine iminosugars were found to inhibit the early biofilm formation of *Pseudomonas aeruginosa* [27]. 

Despite their powerful pharmacological activity, the access of iminosugars to clinics is hampered in many cases by the limited selectivity in vivo, mainly owing to the side inhibition of various off target glycosidases. In order to balance biological activity and toxicity, many structural modifications have been proposed over recent years [28]. Among the most important elements so far identified, *N*-alkylation of iminosugars is known to considerably enhance the target selectivity [12]. On the other hand, changes in the configuration of iminosugar stereocenters have often provided unexpected yet promising results [29]. Particularly l-iminosugars, which were non-superimposable mirror images of the corresponding d-iminosugars, showed higher selectivity towards specific enzymes [21,30,31] and an even more interesting pharmacological potential towards several diseases than their d-enantiomers [20,21,32,33,34]. Among l-iminosugars with a *gluco* configuration, our recent findings highlighted the potential of l-NBDNJ (the enantiomer of the iminosugar drug d-NBDNJ, Zavesca^®^) as a promising and selective new candidate for the combination therapy of Pompe disease [34]. Moreover, l-NBDNJ and its congeners showed interesting pharmacological properties as anti-inflammatory agents in cystic fibrosis [21,22].

**Figure 2:**
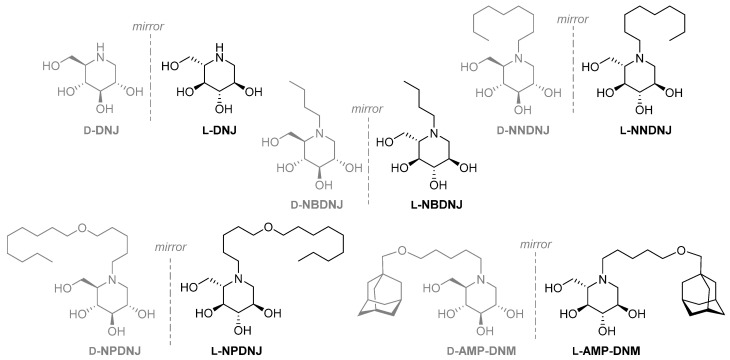
d- and l-deoxyiminosugars studied in this work. DNJ, deoxynojirimycin; NBDNJ, *N*-butyl DNJ; NNDNJ, *N*-nonyl DNJ; NPDNJ, *N*-nonyloxypentyl DNJ; AMP-DNM, *N*-[5-(adamantan-1-ylmethoxy) pentyl] -1-DNJ.

In the present study, we analyzed the inhibition of *S. aureus* growth by L-DNJ and its N-alkylated derivatives (Figure 2) and the effects on the biofilm formation and virulence factors expression of *S. aureus* of *N*-nonyloxypentyl-l-DNJ (l-NPDNJ) (Figure 2), the iminosugar enantiomer showing the highest antibacterial activity against *S. aureus*.

## 2. Results and Discussion

### 2.1. Antimicrobial Activity of D- and L-DNJ and of A Library of Their N-Alkyl Derivatives

The synthesis of unnatural l-DNJ was accomplished exploiting a de novo previously reported methodology [34,35,36]. *N*-alkylation of l-DNJ, as well as of d-DNJ, provided the corresponding *N*-alkylated derivatives as previously described (Figure 2) [21].

To analyze the effect of the small library of deoxyiminosugars in both enantiomeric forms on the growth of *S. aureus* ATCC 29213, the minimum inhibitory concentration (MIC) and minimum bactericidal concentration (MBC) were determined by a broth microdilution assay (Table 1).

Previous studies showed that d-DNJ has antimicrobial activity against *S. mutans* [24,25]. Conversely, DNJ and NBDNJ in both enantiomeric forms were inactive against *S. aureus* ATCC 29213 cells (Table 1, entries 1 to 4). Both enantiomers of NNDNJ (entries 5 and 6) showed very low antimicrobial activity at the MIC value of 1000 μg/mL. d- and l-AMP-DNM (entries 9 and 10) and d-NPDNJ (entry 7) showed poor antimicrobial activity, with MIC values ranging from 256 to 512 µg/mL. The antibacterial activity of l-NPDNJ (entry 8) was found to be the best one in this series, with an MIC value of 128 μg/mL and an MBC value of 256 μg/mL. The data clearly suggest a role of the lipophilicity in the antimicrobial potential of the examined iminosugars, as only the more hydrophobic piperidines showed an inhibitory effect. Differently from previous results [21,34], sugar chirality did not appear to have a major role in the antibacterial activity of the corresponding iminosugars. Even in the case of NPDNJ, only a slight preference for the l-enantiomer was detected. In addition, it should be noted that the antimicrobial activity was lower than that of the marketed drug gentamicin, used as the reference compound (entry 11). The absence of cytotoxicity for all the tested compounds has been previously evaluated [21].

Then, we analyzed the antimicrobial activity of l-NPDNJ against a panel of ten *S. aureus* clinical isolates showing different antibiotic resistance profiles (Table S1). The MIC and MBC values of l-NPDNJ against *S. aureus* clinical isolates were identical to those obtained against the reference *S. aureus* ATCC 29213 strain (Table S2). Moreover, all *S. aureus* clinical isolates and the *S. aureus* ATCC 29213 strain showed an MBC/MIC ratio of 2 for l-NPDNJ (Table S2), thus indicating that this compound exhibited bactericidal activity [37].

**Figure 3:**
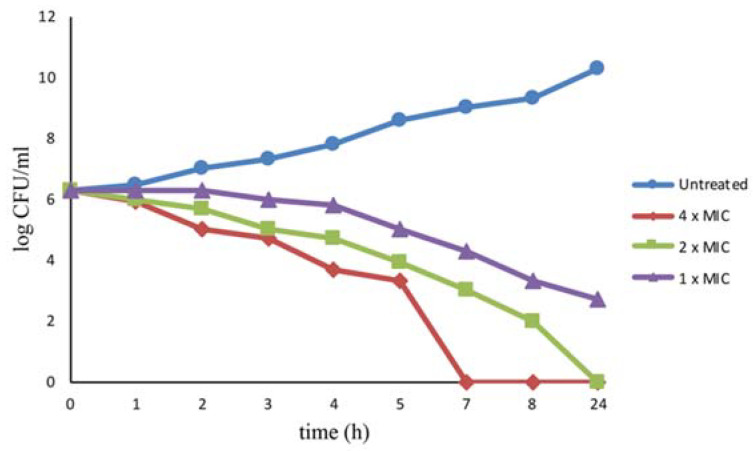
Killing kinetics for *S. aureus* ATCC 29213 following treatment with the l-NPDNJ. Growth kinetics were monitored following exposure to l-NPDNJ at 1× MIC, 2× MIC and 4× MIC.

The bactericidal effect induced by l-NPDNJ was evaluated also by time kill studies. The bacteria were challenged with 1× MIC (128 μg/mL), 2× MIC (256 μg/mL) or 4× MIC (512 μg/mL) of l-NPDNJ and the time course of bacterial growth inhibition was followed by monitoring the viable bacterial counts (Figure 3). 

At the concentration of 512 μg/mL, l-NPDNJ completely killed the strain after incubation for 7 h. No viable *S. aureus* ATCC 29213 cells were recovered after 24-hours exposure at 256 μg /mL (2× MIC). l-NPDNJ at the concentration of 128 μg/mL did not completely kill the strain after incubation for 24 h, although the concentration of the bacteria was reduced to 2 × 10^3^ CFU/mL after l-NPDNJ treatment for 8 h (Figure 3). The time kill kinetic studies confirmed the efficacy of compound l-NPDNJ against *S. aureus*.

In subsequent experiments, we studied whether l-NPDNJ may have combination effects when mixed with other compounds. To define the nature of the interaction (antagonistic, indifferent or synergistic) between enantiomeric species, the combination effect mixing d- and l-NPDNJ was evaluated by a broth microdilution checkerboard assay, using the fractional inhibitory concentration method described by Hall et al. [38]. When both enantiomers were combined in equimolar amounts, a two-fold reduction in the MIC value for both d-NPDNJ (from 256 to 128 μg /mL) and l-NPDNJ (from 128 to 64 μg/mL) was observed, indicating an enhancement of the antimicrobial activity as a consequence of an additive effect by the racemic mixture (fractional inhibitory concentration index (FICI) value = 1). To assess whether l-NPDNJ was able to potentiate or restore the antibacterial activity of currently available antibiotics against *S. aureus*, the iminosugar was evaluated in combinations with gentamicin and oxacillin against the clinical isolate *S. aureus* 00717 [39], which was resistant to both antimicrobials (Table S1). The checkerboard microdilution assay showed that l-NPDNJ markedly lowered the MIC values of gentamicin (from 256 to 8 μg/mL) and oxacillin (from 128 to 1 μg/mL) against the bacterial strain (Table 2). These results demonstrated that the combination of l-NPDNJ with either gentamicin or oxacillin had an additive effect, as the FICI values were 0.5078 and 0.5312, respectively (Table 2). Interestingly, our data revealed that l-NPDNJ was able to restore the efficacy of oxacillin against methicillin-resistant *S. aureus* strains, while improving the antimicrobial activity of gentamicin. 

### 2.2. Effects of l-NPDNJ on Formation of S. Aureus Biofilm

**Figure 4:**
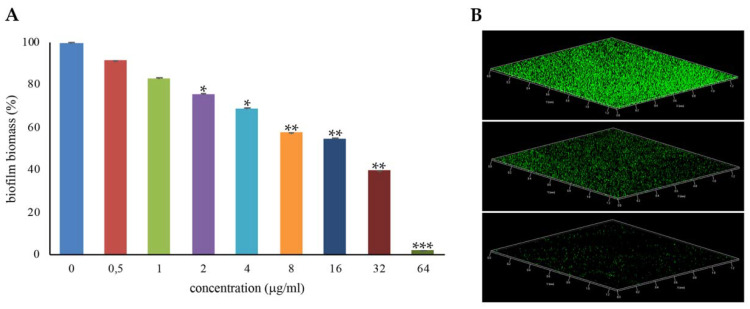
Inhibition of *S. aureus* ATCC29213 biofilm formation with l-NPDNJ. (**A**) Biofilm was quantified after crystal violet staining. Values are presented as means ±SDs. Asterisks indicate statistically significant differences between treated and untreated biofilms (* *p* < 0.05, ** *p* < 0.01, *** *p* < 0.001). (**B**) Confocal laser scanning microscopy (CLSM) analysis of the biofilm formed in the absence (upper panel) or presence of l-NPDNJ at the concentrations of 32 (middle panel) and 64 μg/mL (inferior panel).

Most *S. aureus* strains form biofilms on several biotic and abiotic surfaces, and this greatly contributes to the pathogenicity of this species [5,6]. To assess whether the antimicrobial iminosugar l-NPDNJ affected the biofilm formation of the *S. aureus* ATCC 29213 strain, the biofilm biomass of *S. aureus* ATCC 29213 cells treated with increasing concentrations of l-NPDNJ in static conditions at 37 °C was analyzed using abiotic crystal violet staining. Sub-inhibitory concentrations of l-NPDNJ were able to reduce the biofilm formation of *S. aureus* ATCC 29213 compared with the untreated control in a dose-dependent manner (Figure 4).

In particular, a reduction of 98%, 60% and 45% in the biofilm biomass of *S. aureus* ATCC 29213 was found at the l-NPDNJ concentrations 64, 32 and 16 μg/mL, corresponding to 1/2× MIC, 1/4× MIC and 1/8× MIC, respectively. To determine whether the inhibitory effect on biofilm formation was related to growth inhibition, planktonic growth was measured in the same conditions used in the biofilm assay. At the concentrations tested in the biofilm assay, l-NPDNJ did not affect the planktonic growth of the *S. aureus* ATCC 29213 strain (data not shown).

There are currently only a few papers focusing on the antibiofilm activity of iminosugars. Strus et al. showed the inhibition of *P. aeruginosa* biofilm formation by specific iminosugars, probably owing to the interference with the biosynthesis of extracellular polymeric substances [27]. However, this effect was found to occur only at high concentrations. Similarly, d-DNJ was reported to inhibit adherence and biofilm formation by *S. mutans*, reducing the expression of the glucosyltransferase genes responsible for the sugar-dependent *S. mutans* biofilm formation [24,25,26]. In contrast, pyrrolidine-based hamamelitannin analogues are inactive as antibiofilm agents [40].

In conclusion, sub-MIC values of l-NPDNJ exhibited no bactericidal activity against *S. aureus* but affected *S. aureus* biofilm formation in a dose-dependent manner, inducing a strong reduction in the biofilm biomass. 

### 2.3. Antivirulence Activity of l-NPDNJ in S. Aureus ATCC 29213 

**Figure 5:**
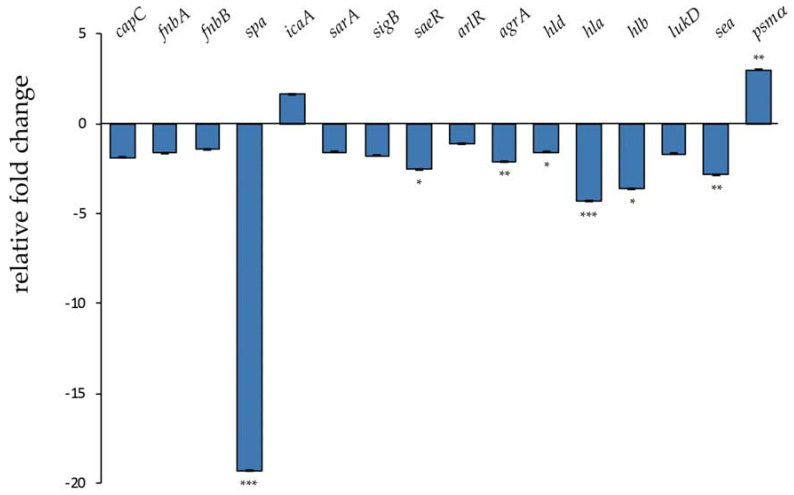
Transcriptional profiling of virulence factor genes in *S. aureus* ATCC 29213 after treatment with l-NPDNJ determined by qRT-PCR. Data were normalized to *rpoB* expression. Fold-changes were calculated using treated versus untreated *S. aureus* cells. Asterisks indicate statistically significant differences between treated and untreated *S. aureus* cells (* *p* < 0.05, ** *p* < 0.01, *** *p* < 0.001). *capC*, capsule biosynthesis protein C; *fnbA*, fibronectin-binding protein A; *fnbB*, fibronectin-binding protein B; *spa*, surface protein A; *icaA*, intercellular adhesion protein A; *sarA*, staphylococcal accessory regulator A; *sigB*, RNA polymerase sigma factor B; *saeR*, response regulator SaeR; *arlR*, response regulator ArlR; *agrA*, accessory gene regulator A; *hld*, delta-hemolysin; *hla*, alpha-haemolysin; *hlb*, beta-haemolysin; *lukD*, pore-forming leucocidin; *sea*, staphylococcal enterotoxin A; *psm*α, alpha phenol-soluble modulin.

Therapeutic agents able to reduce *S. aureus* virulence, especially toxin production, would help to control infections caused by multidrug-resistant strains [10,11]. In order to investigate the effect of l-NPDNJ on *S. aureus* virulence genes [7], the expression levels of 16 virulence genes were measured by reverse transcription real-time PCR (qRT-PCR) in *S. aureus* ATCC 29213, following treatment with l-NPDNJ (Figure 5). 

The transcriptional analysis was performed on samples that were harvested after treatment of *S. aureus* cells (5 × 10^8^ CFU/mL) at a sub-MIC concentration of l-NPDNJ for 3 hours. Untreated *S. aureus* cells grown in the same conditions were used as the control. No growth differences between treated and untreated cells were observed. In this type of experiment, genes with at least a 2-fold difference in the relative transcript levels and with a P value of < 0.05 were considered significant. As shown in Figure 5, the expression of about half of the tested genes was affected by l-NPDNJ treatment. 

The *S. aureus* virulence arises from the combination of a comprehensive array of virulence determinants including surface-associated proteins, exotoxins, enterotoxins and superantigens [8]. The *S. aureus* hemolysins alpha-toxin (Hla), beta-toxin (Hlb) and phenol-soluble modulins (PSMs) all cause the lysis of eukaryotic cells [41,42] and may also contribute to the formation of biofilms [5,43,44]. 

Mounting evidence identifies *S. aureus* toxins as potential targets of anti-virulence therapy [45,46]. In agreement with this, our results showed that the treatment of *S. aureus* cells with l-NPDNJ decreased significantly the expression of the *hla*, *hlb* and *sea* (encoding enterotoxin A) genes (Figure 5). Alpha toxin, a small pore-forming toxin, is a potent secreted cytolysin essential to the development of serious infections, including skin and soft tissue infections, pneumonia and bacteremia [41,47]. Hla is also required for cell–cell interactions during biofilm formation and is employed by *S. aureus* to evade the host immune system [48]. Many molecules can suppress Hla production by *S. aureus* or inhibit the self-assembly of Hla [49]. Beta toxin, a sphingomyelinase toxic for different types of human cells, forms an insoluble nucleoprotein in the biofilm matrix and stimulates biofilm formation in vivo, which is an important process occurring during infective endocarditis [44]. The staphylococcal enterotoxin A is the toxin most frequently reported to cause staphylococcal food poisoning [50]. It has been suggested that SEA may be involved during the early phases of colonization [51]. Our data are in accordance with a recent report, showing that both baicalin and thymol treatments reduced *sea* and *hla* levels [52,53]. 

In contrast with the effect observed on the expression of *hla* and *hlb*, we found that the treatment with l-NPDNJ resulted in the upregulation of the *psm* gene expression. PSMs genes belong to a novel family of toxins and play multiple roles during *S. aureus* pathogenesis, which typically involve blood cell lysis and biofilm development [5,54]. PSMs are small amphipathic peptides that can also disrupt cellular interactions within biofilms, facilitating the structuring and detachment of staphylococcal biofilms [42]. PSMs exhibit cytolytic activity against eukaryotic cells and this effect is diminished in human serum [9]. A recent paper reported low concentrations of both the clindamycin- and secalonic acid D-induced *psm* gene expression in *S. aureus* planktonic cells [55,56]. Overall, the downregulation of the expression of toxin genes observed during treatment with l-NPDNJ and other antimicrobials may reduce the damage of eukaryotic cells and/or biofilm formation of *S. auerus*. 

In addition to the downregulation of toxin genes, l-NPDNJ was effective in decreasing the transcription of the *spa* gene (-19.3-fold), encoding surface protein A, whereas no significant changes were observed in the fibronectin binding protein A (*fnbA)* and B (*fnbB*) gene expressions (Figure 5). Moreover, l-NPDNJ has no effect on the expression of *icaA*, which encodes for a transmembrane glucosyltransferase synthetizing polysaccharide intercellular adhesin (PIA) oligomers [57] (Figure 5). Protein A binds the Fc region of IgG, which may serve to limit infections in vivo [58]. Protein A is also involved in *S. aureus* host colonization inducing aggregation and biofilm development [58,59]. Thus, the decreased *spa* expression observed after l-NPDNJ treatment might contribute to the decrease in biofilm formation. Similarly, the benzimidazole derivative UM-C162 exhibits antivirulence and antibiofilm activities through the downregulation of *hlb*, *spa* virulence-associated genes and the *saeR* response regulator [60]. In addition, the benzimidazole-derived molecule ABC-1 inhibits biofilm formation primarily by reducing the *spa* expression and accumulation of PIA on the cell surface without affecting the transcription of the *icaA* gene [61]. Recently, Hassan et al. [25] showed that d-DNJ downregulates a panel of genes encoding for glucosyltransferases involved in the synthesis of glucans (*gtfC*, *gtfD*, *gbpB*), surface protein (*spaP*) and the quorum-sensing regulatory gene (*comDE*), which are critical for *S. mutans* adherence to the tooth surface and virulence. 

Finally, we observed that the treatment with l-NPDNJ reduced the expression of genes encoding for two global regulators of *S. aureus* virulence, the accessory gene regulator A (AgrA) and response regulator SaeR (Figure 5). The quorum-sensing system *agr* plays a key role in the regulation of *S. aureus* virulence genes [62]. The *agr* operon regulates both biofilm formation and the production of toxins, such as hemolysin (Hla and Hlb), enterotoxins and SpA [63,64,65]. PSMs are positively regulated by the direct binding of AgrA to the its promoter region [66] and negatively by the direct binding of the transcriptional regulator MgrA to the promoter region [67]. The staphylococcal accessory regulator (SarA) and the small RNA Teg41 can also influence the expression of PSMs [42,68]. In most strains of *S. aureus*, the *agr* system activates the transcription of the two-component system SaeRS [69]. SaeRS controls the expression of major virulence genes, including cell wall proteins (such as Spa and FnbA) and secreted toxins (such as HlA and Hlb), probably through direct interaction with these target genes [70,71]. The upregulation of virulence genes by SaeRS facilitates *S. aureus* survival following neutrophil evasion and pathogenesis [72,73]. It is possible that the downregulation of the *agrA* and *saeR* genes could contribute to the downregulation of the other virulence genes tested, reducing the deleterious effects that occur in *S. aureus* infections. 

Overall, our data demonstrate that l-NPDNJ inhibits the expression of important virulence and global regulator genes in *S. aureus*, attenuating probably the virulence of this pathogen. Future studies will be necessary to establish the molecular mechanism responsible for the effect of l-NPDNJ on *S. aureus* planktonic growth and biofilm formation. 

## 3. Materials and Methods 

### 3.1. Chemicals and Reagents

All chemicals and solvents were used with the highest degree purity and without further purification (Sigma-Aldrich, Alfa Aesar, VWR). TLC monitoring was used to follow the reaction course (F254 Merck silica gel plate) by exposure to ultraviolet radiation, iodine vapor and chromic mixture. Intermediates and final products were characterized by NMR analysis (NMR spectrometers: Bruker DRX and Bruker AVANCE 400 MHz). Compound purity was analyzed by elemental analysis (CHNS). Synthesis of iminosugars was accomplished as previously reported [21,34]. Iminosugars were dissolved in ultrapure DNase/RNase-free distilled water to the concentration of 20 mg/mL.

### 3.2. Bacterial Strains and Growth Conditions

*S. aureus* ATCC 29213 and *S. aureus* clinical isolates described by us previously [39,74] were grown overnight at 37 °C on Trypticase soy agar (TSA) with 5% sheep blood. The MIC of oxacillin was determined by a broth microdilution method according to the recommended procedures by the Clinical and Laboratory Standards Institute [75]. Methicillin-resistant *S. aureus* (MRSA) isolates (Table S1) were screened for the presence of staphylococcal cassette chromosome *mec* (SCC*mec*) using the Xpert MRSA kit (Cepheid, Sunnyvale, CA, USA).

### 3.3. Determination of Minimum Inhibitory Concentration (MIC) and Minimum Bactericidal Concentration (MBC)

MIC and MBC values of iminosugars were determined by a broth microdilution method as previously described [76]. Briefly, 180 μL of cation-adjusted Mueller–Hinton broth (CA-MHB) containing serially diluted DNJ derivatives was inoculated with a 5 × 10^5^ colony forming unit (CFU) and incubated at 37 °C for 18–24 h under shaking (300 rpm). The final concentration of the tested compounds ranged from 1000 to 16 μg/mL. Non-treated bacteria were used as controls. All tests were performed in triplicate and repeated three times.

### 3.4. Time Killing Assay

The time-killing kinetics of l-NPDNJ were determined against the *S. aureus* ATCC 29213 strain, as previously described [77]. A bacterial inoculum (approximately 5 × 10^6^ CFU/mL) was added to tubes containing different concentrations of l-NPDNJ (512, 256 and 128 μg/mL), and incubated at 37 °C under shaking (300 rpm). A tube without l-NPDNJ was used as the growth control. A 50 μL aliquot was removed from the bacterial culture at 0, 1, 2, 3, 4, 5, 7, 8 and 24 h and serial 10-fold dilutions of these cultures were plated on TSA for the determination of CFU/mL. All experiments were repeated three times. 

### 3.5. Checkerboard Assay

Tests were carried out using the microbroth checkerboard method according to the previously reported method [38]. Serial dilutions of l-NPDNJ (256–8 μg/mL in sterile water) were prepared and combined with serial dilutions of gentamicin (512–1 μg/mL in sterile water) or oxacillin (256–0.5 μg/mL in sterile water). Subsequently, each of the different concentrations of antibiotics and of l-NPDNJ was added to each well of the microtiter plate containing approximately 5 × 10^6^ CFU/mL of the *S. aureus* isolate 00717. The plates were then incubated at 37 °C for 18–24 h. The combined effects were then determined by calculating the fractional inhibitory concentration (FIC) index as follows: FICI = FIC_A_ + FIC_B_, where FIC_A_ is the ratio of the MIC of the L-NPDNJ combination and the MIC of L-NPDNJ alone and FIC_B_ is the ratio of the MIC of the antibiotic in combination and the MIC of the antibiotic alone. The FIC index is interpreted as synergy (FICI ≤0.5), additive (FICI >0.5 to ≤1.0), indifference (FICI >1.0 to ≤2.0) and antagonism (FICI >2.0) [38,78]. All experiments were repeated three times.

### 3.6. Biofilm Assay

Biofilm formation was examined using a crystal violet (CV) staining assay and confocal laser scanning microscopy (CLSM), according to the previously reported method [79]. The 0.5 McFarland of bacterial cultures was diluted 1:100 in TSB supplemented with 0.5% glucose. Then, 5 × 10^6^ cells/mL was transferred into a 96-well flat-bottomed polystyrene microtiter plate containing 100 μL of scalar doses of l-NPDNJ (ranging from 64 to 0.5 μg/mL and 64 to 32 μg/mL in CV and CLSM assays, respectively) and incubated at 37 °C for 24 h. Non-treated bacteria were incubated with 100 μL of broth and used as the control. The culture supernatant was gently discarded, the wells were washed twice with phosphate-buffered saline (PBS) 1× pH 7.4 and the biofilms were stained with 200 μL of 0.1% crystal violet for 15 min. The wells were washed twice with PBS 1X, and dye was re-eluted with 100% ethanol. The absorbance was measured at 595 nm using a microplate reader (Bio-Rad Laboratories S.r.l.). All data points are expressed as means ± SDs of three separate experiments performed in triplicate.

### 3.7. RNA Purification and Real-Time RT-PCR

*S. aureus* ATCC 29213 cells were grown at 37 °C at 200 rpm to the exponential phase (OD_600_ = 0.4) and were subsequently split into two tubes and incubated in the presence or absence of l-NPDNJ at the concentration of 64 μg/mL for a further 3 h. Total RNA was isolated from three independent cultures according to the previously reported method [80]. The cDNA was synthesized using QuantiTect Reverse Transcription Kit (Qiagen), according to the manufacturer’s protocol. Real-time RT-PCR assays were performed by using a SYBR Green master mix (Applied Biosystems) as previously described [81]. The expressions of target genes were normalized using the *rpoB* gene as the housekeeping gene. The fold-change of the gene expression level was calculated using the 2−^ΔΔCT^ method [82]. All experiments were performed three times in triplicate. The primers used in the qRT-PCR experiments are reported in Table 3.

### 3.8. Statistical Analysis

All statistical analyses were carried out using GraphPad Prism version 8.0 for Windows (GraphPad Software, San Diego, CA, USA). All experiments were performed at least three times and the results are shown as means ± SD. Differences between mean values were tested for significance by performing two-tailed Student’s t-tests. A *P* value < 0.05 was considered to be statistically significant.

## 4. Conclusions

We have herein reported one of the earliest studies on the antimicrobial properties of d- and l-iminosugars, with a special focus on their effects on the growth, biofilm formation and virulence factor expression of *S. aureus*. Our results have brought to identify in the iminosugar l-NPDNJ a new antimicrobial drug against *S. aureus* because of its antibacterial activity at 128 μg/mL MIC and bactericidal effect at 2 × MIC. Noteworthy, combination effects were observed when l-NPDNJ was administered with the marketed antimicrobial drugs gentamicin and oxacillin, leading to an enhancement or a restoration of the antibacterial activity, respectively. Further, sub-MIC concentrations of l-NPDNJ were able to inhibit the *S. aureus* biofilm formation. Even more intriguingly, an addictive effect was observed when mixing equimolar amounts of the enantiomeric iminosugars d- and l-NPDNJ, although in this case, the enhancement of the antimicrobial activity was less marked. Moreover, l-NPDNJ was found to inhibit the expression of virulence and global regulator genes. Overall, our data suggest that l-NPDNJ and most generally lipophilic l-iminosugars may represent new candidates for the anti-virulence therapy of *S. aureus*. Future studies will be necessary to evaluate the therapeutic use of l-NPDNJ during *S. aureus* infections.

## Figures and Tables

**Table 1:** Minimum inhibitory concentration (MIC) (μg/mL) and minimum bactericidal concentration (MBC) (μg/mL) values of d- and l-DNJ and their *N*-alkyl derivatives against *S. aureus* ATCC 29213.

Entry	Compound	MIC	MBC
1	d-DNJ	>1000	>1000
2	L-DNJ	>1000	>1000
3	d-NBDNJ	>1000	>1000
4	l-NBDNJ	>1000	>1000
5	d-NNDNJ	1000	>1000
6	l-NNDNJ	1000	>1000
7	d-NPDNJ	256	1000
8	l-NPDNJ	128	256
9	d-AMP-DNM	256	>1000
10	l-AMP-DNM	512	>1000
11	Gentamicin	1	2

Abbreviations: MIC, minimum inhibitory concentration; MBC, minimum bactericidal concentration; DNJ, deoxynojirimycin.

**Table 2:** Additive effect of l-NPDNJ with antibiotics against *S. aureus* isolate 00717.

Bacterial Strain	Combination	MIC^a^ (μg/mL)	MIC^c^ (μg/mL)	FICI
*S. aureus* 00717	l-NPDNJ/oxacillin	128/128	64/1	0.5078
	l-NPDNJ/gentamicin	128/256	64/8	0.5312

MIC^a^, MIC of one sample alone; MIC^c^, MIC of samples in combination; FICI, fractional inhibitory concentration index.

**Table 3:** Gene target list and oligonucleotide sequences.

Gene	Forward Primer (5’-3’)	Reverse Primer (5’-3’)	Reference
*agr*A	TGCGAAGACGATCCAAAAC	TTTAGCTTGCTCAAGCACCTC	[39]
*arlR*	AGTTGCTGGGCTTGATTACG	ATCCTTTTGTGGCTGACGAC	this study
*cap*C	CATCCAGAGCGGAATAAAGC	CGGAAATACCCGCTAATGAC	[39]
*fnb*A	AAGCACAAGGACCAATCGAG	ACGCCATAATTACCGTGACC	this study
*fnb*B	GAACATGGTCAAGCACAAGG	ACGCCATAATTACCGTGACC	[39]
*hla*	TCTTGGAACCCGGTATATGG	AGCGAAGTCTGGTGAAAACC	[39]
*hlb*	GTGCCAAAGCCGAATCTAAG	ATCAGCGCGTTTATATTGTCC	[39]
*hld*	AAGGAAGGAGTGATTTCAATGG	TTTGTTCACTGTGTCGATAATCC	[79]
*icaA*	ACGCAGCAGTAGTTCTTGTCG	TGACCATGTTGCGTAACCAC	this study
*luk*D	GTACTTAAGGCAGCCGGAAAC	CGCCCCAATAAAACTGTGAG	[39]
*psmα*	TCAAAAGCTTAATCGAACAATTCAC	AATGGCCCCCTTCAAATAAG	[79]
*rpo*B	ACAACCACTTGGCGGTAAAG	ATGCTTCAAGTGCCCATACC	[39]
*sae*R	CCAAGGGAACTCGTTTTACG	ACGCATAGGGACTTCGTGAC	[39]
*sar*A	TTGCTTTGAGTTGTTATCAATGG	CAATACAGCGAATTCTTCAAAGC	[79]
*sea*	ATTGCCCTAACGTGGACAAC	TGCTCCCTGCAATTCAGAC	[39]
*sig*B	TGATCGCGAACGAGAAATC	ATTGCCGTTCTCTGAAGTCG	[39]
*spa*	AGATGACCCAAGCCAAAGTG	CTTTCGGTGCTTGAGATTCATT	[39]

## Supplementary Materials

The following are available online at https://www.mdpi.com/2079-6382/9/6/362/s1, Table S1: Antibiotic susceptibility of clinical isolates used in this study, Table S2: MIC (μg/mL) and MBC (μg/mL) values of L-NPDNJ for clinical isolates used in this study.
