# Supplementary material for: N-Nonyloxypentyl-l-Deoxynojirimycin Inhibits Growth, Biofilm Formation and Virulence Factors Expression of Staphylococcus aureus
Source: Antibiotics (Basel). 2020 Jun 26;9(6):362. doi: 10.3390/antibiotics9060362 (PMC7344813; doi:10.3390/antibiotics9060362)
Supplement: Supplementary file 1 [file antibiotics-09-00362-s001.pdf]

Supplementary Materials

Table S1. Antibiotic susceptibility of clinical isolates used in this study.

Strain	Oxacillin	Levofloxacin	Erythromycin	Linezolid	Daptomycin	Teicoplanin	Vancomycin	Tetracycline	Fosfomycin	Rifampicin	Clindamycin	Tigecycline	Gentamicin	Ciprofloxacin
S. aureus ATCC 29213	0,125 (S)	0,125	<0,5 (S)	0,5 (S)	ND	ND	1 (S)	0,5 (S)	1 (S)	0,016 (S)	<0,125 (S)	ND	0, 5 (S)	ND
S. aureus 00717	128 (R)	>4 (R)	>4 (R)	2 (S)	0,25 (S)	<0,5 (S)	1 (S)	<1 (S)	>64 (R)	>2 (R)	0,25 (R)	<0,12 (S)	256 (R)	ND
S. aureus 60338	>2 (R)	ND	>2 (R)	2 (S)	<0,5 (S)	<=0,5 (S)	<=0,5 (S)	<=0,5 (S)	>64 (R)	<0.25 (S)	0.25 (R)	<=0,25 (S)	<=1 (S)	>4 (R)
S. aureus 60419	<=0,25 (S)	ND	>2 (R)	1 (S)	<0,5 (S)	<=0,5 (S)	1 (S)	>2 (R)	<16 (S)	ND	0,25 (R)	0.5 (S)	>4 (R)	>4 (R)
S. aureus 61030	2 (R)	ND	>2 (R)	1 (S)	<0,5 (S)	<0,5 (S)	<=0,5 (S)	<=0,5 (S)	<16 (S)	<0.5 (S)	0,25 (R)	<=0,25 (S)	<=1 (S)	>4 (R)
S. aureus 61035	>2 (R)	>4 (R)	>4 (R)	2 (S)	0,5 (S)	<=0,5 (S)	<=0,5 (S)	<1 (S)	>64 (R)	<0.03 (S)	0,25 (R)	0,25 (S)	<=0, 5 (S)	>4 (R)
S. aureus 61050	>2 (R)	<=0,12 (S)	>4 (R)	2 (S)	0,25 (S)	<=0,5 (S)	<=0,5 (S)	>8 (R)	ND	<0.03 (S)	0,25 (R)	<=0,12 (S)	<=0, 5 (S)	ND
S. aureus 61486	>2 (R)	>4 (R)	1 (S)	2 (S)	0,5 (S)	<=0,5 (S)	<=0,5 (S)	<1 (S)	>64 (R)	<0.03 (S)	0,25 (R)	0,25 (S)	<=0, 5 (S)	>4 (R)
S. aureus 62482	>2 (R)	ND	>2 (R)	2 (S)	1 (S)	<=0,5 (S)	1 (S)	>2 (R)	<16 (S)	ND	0,25 (R)	<=0,25 (S)	>4 (R)	1 (S)
S. aureus 63906	>2 (R)	>1 (R)	0,5 (S)	2 (S)	1 (S)	1 (S)	1 (S)	<=0,5 (S)	>64 (R)	<0.25 (S)	<0,25 (S)	<=0,25 (S)	<=1 (S)	>4 (R)
S. aureus 64428	<=0,25 (S)	>1 (R)	>4 (R)	2 (S)	1 (S)	<=0,5 (S)	1 (S)	<1 (S)	<16 (S)	<0.03 (S)	0,25 (R)	<=0,12 (S)	<=0, 5 (S)	>4 (R)

The MIC values were expressed as μg/mL. MICs were determined by BD Phoenix PMIC/ID-88 panel. The categorical interpretations were as follows: S, susceptible; R, resistant; ND, not determined.

Table S2. MIC (μg/mL) and MBC (μg/mL) values of L-NPDNJ for clinical isolates used in this study.

Strain	MIC	MBC
S. aureus ATCC 29213	128	256
S. aureus 00717	128	256
S. aureus 60338	128	256
S. aureus 60419	128	256
S. aureus 61030	128	256
S. aureus 61035	128	256
S. aureus 61050	128	256
S. aureus 61486	128	256
S. aureus 62482	128	256
S. aureus 63906	128	256
S. aureus 64428	128	256
